# Making and revising predictive inferences during Chinese narrative text reading: Evidence from an electrophysiological study

**DOI:** 10.3389/fpsyg.2022.1061725

**Published:** 2023-01-13

**Authors:** Fei Xu, Lin Fan, Lingyun Tian, Lulu Cheng

**Affiliations:** ^1^School of Humanities and Foreign Languages, Qingdao University of Technology, Qingdao, China; ^2^Research Institute of Foreign Languages, Beijing Foreign Studies University, Beijing, China; ^3^School of Foreign Languages, Harbin University of Science and Technology, Heilongjiang, China; ^4^National Research Centre for Foreign Language Education, Beijing Foreign Studies University, Beijing, China; ^5^School of Foreign Studies, China University of Petroleum (East China), Qingdao, China; ^6^Shanghai Center for Research in English Language Education, Shanghai International Studies University, Shanghai, China

**Keywords:** predictive inference revision, predictive inference alternatives, activation levels, Chinese narratives, contextual constraints

## Abstract

The current study employed the event-related potential (ERP) technique to investigate predictive inference revision during Chinese narrative text reading among Chinese native speakers. Experiment 1 studied predictive inference revision by ensuring high contextual constraints for activation of the primary predictive inferences. Experiment 2 inspected the effects of the weaker inference alternatives on the revision process. Longer reading time and less positive mean average amplitude with two subcomponents of P300 (P3a and P3b) in the revise condition suggest that readers could detect inconsistent information and disconfirm the incorrect predictive inferences. However, they have difficulties in either integrating the alternative predictive inferences (N400) or revising the incorrect ones (P600), especially when the alternatives are of weaker activation levels. This study supports the Knowledge Revision Components (KReC) framework by verifying remaining activation of the disconfirmed primary inferences and extends it by considering effects of competitive alternatives on the predictive inference revision process.

## 1. Introduction

The predictive inference is concerned with readers’ anticipation of events, characters, causal consequences, or other concepts likely to appear later in the text (Graesser et al., 1994). Currently, many researchers tend to consider prediction and predictive inferences as the same thing (e.g., Hawelka et al., 2015; Ferreira and Chantavarin, 2018), while prediction in the current trends focuses more on pre-activation of specific words in a graded fashion through activation of orthographic, phonological, syntactic and conceptual information (e.g., DeLong et al., 2005; Brothers et al., 2020; Kuperberg et al., 2020). For predictive inferences, previous studies concentrated on the understanding of their mechanisms and influencing factors such as textual constraint (e.g., Faust and Kravetz, 1998; Lassonde and O’Brien, 2009), availability of textual information (e.g., Keefe and McDaniel, 1993; Murray et al., 1993), working memory capacity (e.g., Liderholm, 2002; Virtue et al., 2006), reading goals (e.g., van den Broek et al., 2001; Virtue and Joss, 2017), and alternative predictive inferences (e.g., Weingartner et al., 2003; Cranford and Moss, 2019).

Among the very few studies concerning the effects of alternative predictive inferences on the generation and encoding of predictive inferences (e.g., Klin et al., 1999a, 1999b; Weingartner et al., 2003; Zhu et al., 2011; Cranford, 2016; Cranford and Moss, 2019), there have been discrepancies in the possible influences of the alternatives. Some concluded that predictive inferences could not be activated due to the interferences from the alternatives (e.g., Klin et al., 1999a) while others held that predictive inferences could be activated and encoded into readers’ mental representation in spite of the availability of alternative predictive inferences (e.g., Weingartner et al., 2003; Cranford and Moss, 2019). More importantly, prior research failed to pay sufficient attention to the situation in which the originally generated predictive inferences were considered as incorrect and disconfirmed by new information. Revision of predictive inferences happens from time to time since not all expectancies from readers could perfectly match the follow-up information. Though predictive inference revision is essential for achieving consistency in the situation model construction, few investigations have been conducted to reveal its processing mechanisms (e.g., Wright and Newhoff, 2002; Nahatame, 2014; Pérez et al., 2015, 2019).

Moreover, there have been disagreement on whether the inconsistent predictive inferences could be suppressed and disconfirmed. Some found that readers could hardly suppress the contradictory predictive inferences (e.g., Potts et al., 1988) while others believed disconfirmation and revision of the improper predictive inferences could happen (e.g., Wright and Newhoff, 2002; Iseki, 2006). Still others found that many factors, such as readers’ working memory (e.g., Pérez et al., 2015, 2016), their cognitive control abilities (e.g., Pérez et al., 2019), and their second language proficiency (e.g., Nahatame, 2014; Pérez et al., 2019) could exert influences on whether readers could revise the incorrect predictive inferences or not. More importantly, even though readers could revise the initially correct predictive inferences, it does not necessarily mean that the now inconsistent predictive inferences could be replaced completely. According to the empirical results from some studies, the disconfirmed predictive inferences could not only be suppressed but also be deleted and replaced by new ones that were consistent with the current situation model (e.g., Nahatame, 2014; Pérez et al., 2015, 2016, 2019). However, studies and theoretical framework in the field of knowledge revision indicate that the disconfirmed information could possibly stay in the working memory and be reactivated whenever the incoming information comes to support the originally encoded information (e.g., Rapp and Kendeou, 2007; Kendeou and O’Brien, 2014), suggesting potential difficulties in removing the incorrect predictive inferences from readers’ working memory.

To our knowledge, there have been few investigations into the possible influences of alternative predictive inferences on the predictive inference revision process. In the paradigm constructed by Pérez et al. (2015), the first three sentences of the short story, or the introduction, could yield at least two predictive inferences, both of which are plausible with one being more probable. The less probable predictive inferences, termed as the alternative predictive inferences, will be supported by information in the critical sentences in the revise condition, thus contradicting the primarily more probable predictive inferences. In most cases, readers could make more than one predictive inferences, based on the contextual information and their background knowledge. Therefore, the predictive inference revision is essentially based on the competition between the preliminary predictive inferences and the alternative ones. As a result, the availability and activation levels of alternative predictive inferences could quite possibly exert influences on the disconfirmation and revision of the primarily drawn predictive inferences.

Among the very few studies concerning the predictive inference revision procedure, some have employed the event-related potential (ERP) technique (e.g., Pérez et al., 2015). These studies were based on the context-updating theory suggesting that P300 and its components may reflect the inference revision process (Polich, 2003, 2007). According to the above theoretical framework, there are two subcomponents of P300, namely P3a and P3b. The P3a is a central-frontal positivity which becomes very evident when the incoming new information is not consistent with the current representation and is evaluated as new. The P3b is a temporo-parietal positivity, which is found when the context of the incoming information involves updating the outdated information. Therefore, P3a is considered as reflecting the mechanisms of attentional control when new information appears (see Friedman et al., 2001; Pérez et al., 2015). P3b is considered to reflect the processing capacity of updating the once-activated but no-longer-relevant information in a revision process (Kok, 2001; Pérez et al., 2015). Therefore, P3a serves as an index of a top-down, stimulus-driven process taking place in the frontal areas while P3b is supposed to be an index of a bottom-up updating process taking place in the parietal areas (see Polich, 2003). However, abundant evidence suggested the close relationship between the P3b and the disconfirmation of an expectation (see Van Petten and Luka, 2012 for a review). As a result, it is more proper to consider P3b as the disconfirmation of the incorrect predictive inferences instead of the revision. Another ERP component closely related to the current study is the N400. N400 has functionally been interpreted as reflecting semantic integration (e.g., Brown and Hagoort, 1993; Brown et al., 2000; Hagoort et al., 2004), lexical retrieval (Kutas and Federmeier, 2000; Lau et al., 2008; Kutas and Federmeier, 2011), or both integration and retrieval on more recent “hybrid” accounts (Baggio and Hagoort, 2011; Lau et al., 2016; Nieuwland et al., 2020). In the field of predictive inference revision research, one study took the reduction in the mean amplitude data of the N400 as indicating the successful integration of the alternative predictive inferences (Pérez et al., 2015). In another study, the N400 was taken as an index reflecting the processing cost of predictive inference revision and alternative predictive inference integration (Pérez et al., 2019). However, there has been little evidence for costs of failed predictions on the N400 (Van Petten and Luka, 2012, 180). As a result, it is more proper to consider the N400 as an index reflecting the integration of the alternative predictive inferences. If neither subcomponents of the P300 nor the N400 could be reflexive of the revision process, which ERP component could serve as a proper index of it? The existing literature considers the modulation of the amplitude of P600 to be connected with structural integration, difficulties to update an initial interpretation, semantic or syntactic reanalysis, increased demands on revision, updating, or conflict-monitoring/resolution processes (e.g., Boudewyn et al., 2015; Pérez et al., 2020). However, there has been evidence of P600 being known as the index of re-analysis or checking. Specifically, the P600 elicited by syntactic errors and some varieties of semantic errors has been widely accepted as reflecting re-processing costs, arising from either reviewing a prior context to determine what goes wrong or if the problem might be repaired. This kind of re-processing might indicate that a problem is detected in an attempted integration (Van Petten and Luka, 2012). Moreover, though previous studies of inferential semantic revision have not reported the P600 (e.g., Pérez et al., 2015, 2016, 2019), the P600 was regarded as an index relevant to semantic revision (e.g., Pérez et al., 2020). Different from the P600s arising from syntactic violation, the semantic P600s have been incurred by re-analysis, “re-attending,” or prolonged analysis of problematic sentences (see review by van Petten and Luka, 2012). In the current study, the P600 is expected to reflect updating or revision of the incorrect primary predictive inference concepts.

There are basically two aims for the current study. On the one hand, it tries to add evidence to revision of predictive inferences when the initially generated predictive inferences supported by high contextual constraints have become inconsistent with new incoming information. On the other hand, it studies the potential influences of alternative predictive inferences of comparatively weaker activation levels on the revision process. The two experiments, following a similar experimental paradigm to Pérez et al. (2015), address the effects of activation levels of competitive predictive inference alternatives on the predictive inference revision process while ensuring high contextual constraints for the on-line activation of the primary predictive inferences. Experiment 1 studies whether readers could fulfill the predictive revision process in a more strictly controlled high-constrained context to ensure the successful activation of primary predictive inferences. Experiment 2 maintains high contextual constraints while controlling the alternative predictive inferences at low-activation levels, thus low competitiveness against the primary ones, to study the effects of the later on the revision procedure.

## 2. Experiment 1

### 2.1. Method

#### 2.1.1. Participants

Thirty-one Chinese native speakers from one university were paid for participating in the experiment. All had normal or corrected-to-normal vision, and none had any language disorder, neurological disorder, or major head injury diagnosed to have long-term side effects. All of them signed the written informed consent before the experiments and received renumeration after finishing the experiment. Data from eight participants were discarded: four due to technical failures, two due to blinking artifacts and two due to low accuracy rate in answering comprehension questions. The remaining 23 participants (10 males and 13 females, *M*_age_ = 22.04, range 19–26, *SD* = 2.31) were included at last for analysis. All participants were right-handed as assessed by the Edinburgh Handedness Inventory (Oldfield, 1971), with a mean laterality of 0.90 (range 0.65–1, *SD* = 12.43) indicating right-handedness. Participants were also engaged in a conventional Digit Span Forward (*M* = 9.22, range 6–12, *SD* = 1.35) with a total score of 12 and Backward (*M* = 6.96, range 3–11, *SD* = 1.77) with a total score of 10 for assessing working memory (Gong, 1983), indicating high levels of working memory for participants.

#### 2.1.2. Materials

Ninety-three (three for practice and 90 for experiment) five-sentence short Chinese narrative texts were prepared. The themes of these passages were all about typical and everyday experiences. A sample material is shown in Table 1. The first three sentences in Experiment 1 provided strong contextual constraints for activating the primary predictive inferences. There were three versions for Sentence 4 (hereafter referred to as the critical sentence). One version was a neutral one in which the critical sentence continued the introduction without providing support for or disconfirming the initially generated predictive inferences elicited from the introduction. The no revise version was consistent with the primary inferences. The revise version contained information that was inconsistent with the primary predictive inferences and prompted participants to revise the now-inconsistent predictive inferences. Reading times for the critical sentences was recorded. Sentence 5 (hereafter referred to as the ERP sentence) contained a disambiguating word at the very end. The disambiguating word was always consistent with the alternative predictive inferences as further supported by the critical sentence in the revise condition but was inconsistent with the primary predictive inferences elicited from the introduction. The target words were action verbs of two Chinese characters with similar word frequencies and the number of strokes. The target words representing two plausible predictive inferences were gained from a norming study. They were all two-character Chinese verbs that were provided by 22 participants who did not participate in the formal experiment. These participants were of similar ages and had similar educational background with participants in the predictive inference revision experiment. They were requested to infer what might happen next right after reading the introduction and to provide the most probable verbs after reading the introduction of each passage in the first section. The most frequently raised verbs were chosen as the first target concepts representing the preliminary predictive inferences. The second target concepts were also from the procedure to represent the alternative predictive inferences to continue the story in a revise condition. The most frequently raised target verbs to continue the story were then subject to appraisals in a probability judgment test on 7-point Likert-type Scale and a multiple-choice task. There was no significant difference between the word frequencies of the target words representing the predictive inference concepts initially generated (*M* = 17.39, *SD* = 27.94) per million and the disambiguating word (*M* = 17.77, *SD* = 28.95) per million, according to SUBTLEX-CH: Chinese Word and Character Frequencies Based on Film Subtitles from Cai and Brysbaert (2010), *t*(89) = −0.09, *p* = 0.93. There was no significant difference between the number of strokes for the first targets in the primary predictive inferences (*M* = 17.28, *SD* = 4.64) and the disambiguating words (*M* = 16.59, *SD* = 3.89), *t*(89) = 1.08, *p* = 0.29, either. Altogether six different questionnaires with three sections of the norming study were constructed to ensure sufficiently high contextual constraints for the initially generated predictive inference concepts following the introduction part (Sentences 1–3) and strong enough contextual constraints for the alternative predictive inference concepts after the critical sentence in the revise condition (Sentences 1–4). At the end of each narrative text, a comprehension question sentence was presented to encourage participants to read the materials carefully with good comprehension of the contents. The comprehension questions were based on the details from the introduction part of the narrative stories and equally distributed across Sentences 1–3. These questions were not directly related with either the initial predictive inferences or the revised ones. Half of the questions required a “yes” response and the other half a “no” response. An equal number of 12 Chinese characters were contained in each of the five sentences and the comprehension questions in order to avoid extra horizontal electrooculogram activities and artifacts.

**Table 1:** Sample material used in Experiment 1.

Introduction	bias猎杀(*Liè Shā*)	男人顺着狮子的脚印跟过来。 一头母狮子进入了他的视野。 他下意识地摸了一下带的枪。
	*English translation* bias *hunting*	*English translation*
		The man followed footsteps of the lion here.
		A female lion came into his view.
		He subconsciously touched the gun he carried.
Neutral	母狮子身后跟了几只小狮子。	
	*English translation*	
	Several small lions walked behind the female lion.	
No revise	他下定决心把握好这次机会。	
	*English translation*	
	He was determined to grasp this chance.	
Revise	他拿出相机对准了这头狮子。	
	*English translation*	
	He took out a camera and aimed at the lion.	
ERP sentence	他连续按动快门给狮子拍照(*Pāi Zhào*)。	
	*English translation*	
	He continuously clicked the shutter for photographing the lion.	
Comprehension question	这个男人看到了一头狮子吗?	
	*English translation*	
	Did the man find a lion?	

#### 2.1.3. Procedure

Sentences 1–4 were presented one at a time. Participants were instructed to rest their right thumb on a line-advance key, their right index finger on a “z” key, and their left index finger on a “m” key. Each trial began with a cross “+” in the middle of the screen with a duration of 750 ms. When participants were ready to read a passage, they pressed the line-advance key. Each press of the key erased the current line and presented the next line. The comprehension times was measured as the time between key presses. Each participant was instructed to read at a comfortable, normal reading pace. The reading time of the critical sentence in three conditions (namely, neutral, no revise, and revise) was recorded. The ERP sentence was presented word by word with a fixed stimulus-onset asynchrony (SOA) of 300 ms per word (the interval between the onset of the last context word and the onset of the target word), with an interval of 100 ms between two words. In addition, there was a delay of 700 ms after the disambiguating word (or the ERP word) to ensure that the electrophysiological activities were recorded during a sufficiently long time-window (SOA = 1,000 ms). Each Chinese word presented contained no more than three Chinese characters to avoid extra ocular movements. Participants were required to try not to blink during the presentation of words in the ERP sentence. At the end of each trial, participants were presented with a true/false comprehension question. Participants pressed the designated true or false key to give a response.

**Figure 1:**
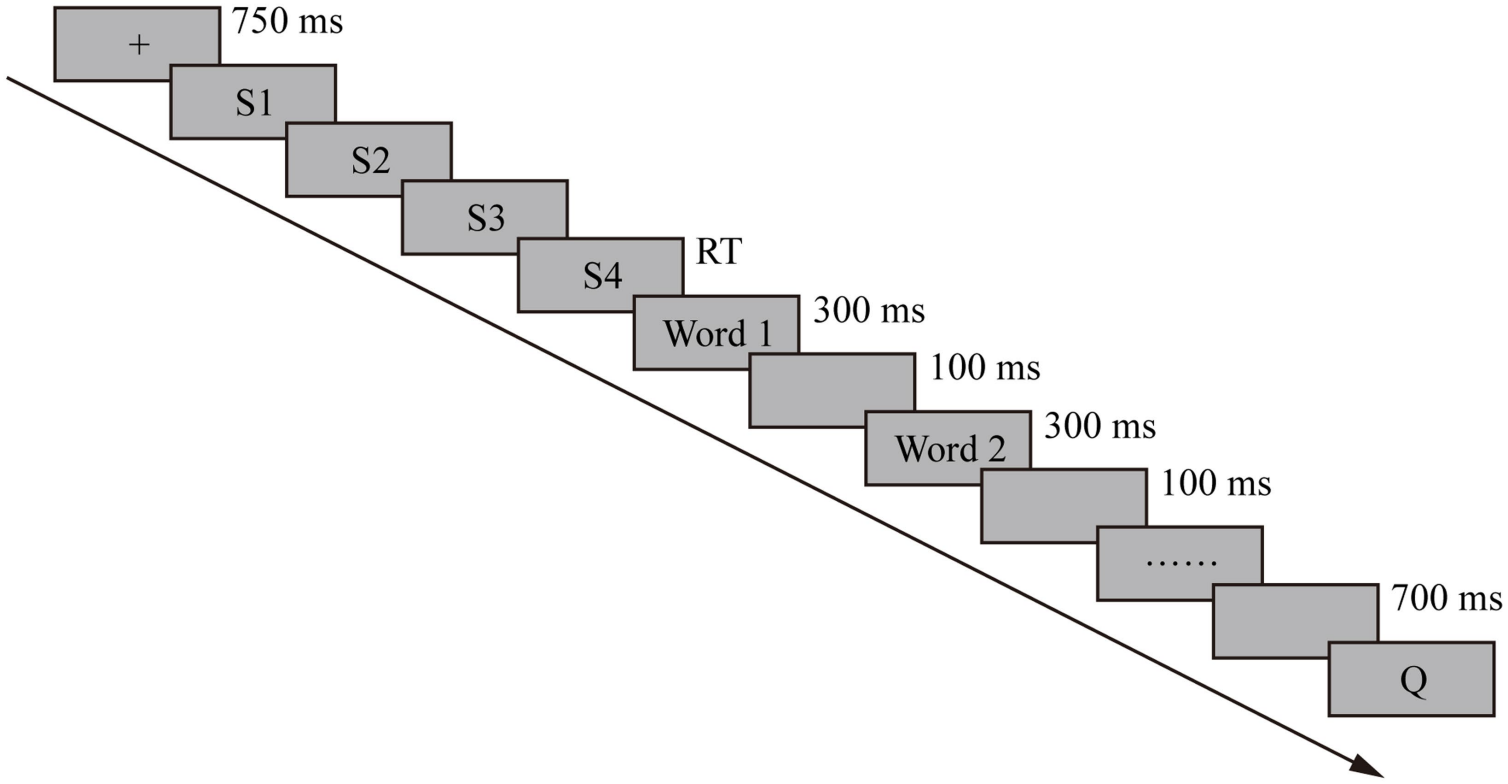
An overview of a trial in Experiment 1.

The 90 sets of experimental texts (in three conditions) were divided into three versions. Each of the 90 experimental texts of a version was presented to every participant only once in one of the three conditions which had been counterbalanced across participants. The task was administered in three blocks, keeping 10 texts in each condition per block (altogether three blocks). The same number of participants were engaged in each condition, and the presentation of texts was randomized within each block. A practice of three trials with no less than 90% accuracy rate ensured that participants had understood and followed the instructions presented on the screen at the very beginning of the experiment. The whole procedure lasted for approximately an hour. Participants were requested to rest for two times according to the experimental design and they could rest between any of the two key presses in the experiment. Experimental procedures of Experiment 1 illustrated by a sample trial is shown in Figure 1.

All tasks were programmed and presented by the E-Prime software (Schneider et al., 2002) and administered on a 19-inch. CRT video monitor (refresh rate = 75 HZ). Based on the 10–20 system, 64 channel electroencephalogram (EEG) was recorded using Ag-AgCl electrodes mounted on an elastic cap (NeuroScan Inc., Herndon, VA, United States). EEG signals were continuously digitized on-line with a continuous sample rate of 1,000 Hz. The default reference electrode worked as referential signals. Ocular movements and blinks were also collected by two pairs of channels. The first was the vertical electrooculogram situated in the left eye of the participant, with one electrode supra and another infraorbitally to measure blink artifact. The other one was the horizontal electrooculogram placed in the external canthi, with one electrode on the left and another on the right side to register eye movements. Impedances were kept below 5 kΩ. The EEG data were analyzed with Curry 8.0 software and was filtered off-line with a band-pass filter (0.1–30 Hz). Artefacts caused by eye movements and eye blinks were eliminated. Bad channels were interpolated. Trials with artifacts were rejected (1.5%) with potentials exceeding ±100 μv. With reference to studies of Pérez et al. (2015, 2019), epochs with an interval between-200 ms (pre-stimulus) and 800 ms (post-stimulus) with respect to the presentation of the target word or the disambiguating word were averaged and analyzed. Baseline correction was applied using the average EEG activity in the 200 ms preceding the onset of the target as a reference signal value. Separate ERPs averages were developed for each condition and for each participant. Individual averages were re-referenced off-line to the average of left and right mastoids. Six regions of interest (ROI) in the current study were chosen out of the 64 electrodes, following the criteria of Pérez et al. (2015, 1112; 2019, 937). These criteria include the symmetry between hemispheres and the same number of electrodes (five sites). The five sites of electrodes are the left frontal (LF), including F1, F3, F5, FC3, and FC5; the right frontal (RF), including F2, F4, F6, FC4, and FC6; the central (C), including C1, C2, CZ, FCZ, and CPZ; the left parietal (LP), including P1, P3, P5, CP3, and CP5; the right parietal (RP), including P2, P4, P6, CP4, and CP6; and the occipital (O) including O1, O2, POZ, PO3, and PO4.

#### 2.1.4. Statistical analysis

Statistical analyses of 23 participants are reported for all trials. Since comprehension questions always corresponded to contents of the first three sentences, they did not affect either the reading times of the critical sentence or the disambiguating word for ERPs. *T*-test analysis confirmed that there were no differences between the reading times of samples with correct responses only and those of the whole sample, *t*(22) = −1.32, *p* = 0.20. As a result, both correct and incorrect responses were included for analysis for both behavioral and electrophysiological analyses.

The behavioral analysis of the predictive inference revision task was conducted on reading times (in milliseconds) for the critical sentence. To minimize outliers, the data that were 3 standard deviations away had been removed from the analyses. All analyses included the between-subjects variables of counterbalanced list. An alpha level of 0.05 was set to determine the significance. All effect sizes were reported in terms of partial eta squared for ANOVAs. For all analyses reported, F1 refers to by subject analyses and F2 refers to by item analyses. Accuracy was calculated by finding the average percent correct for each condition. Accuracy was quite high in all conditions (averaging at 94.52%), close to ceiling and not significantly differing across conditions or participants [*F*(2, 66) = 2.92, *p* = 0.06].

**Figure 2:**
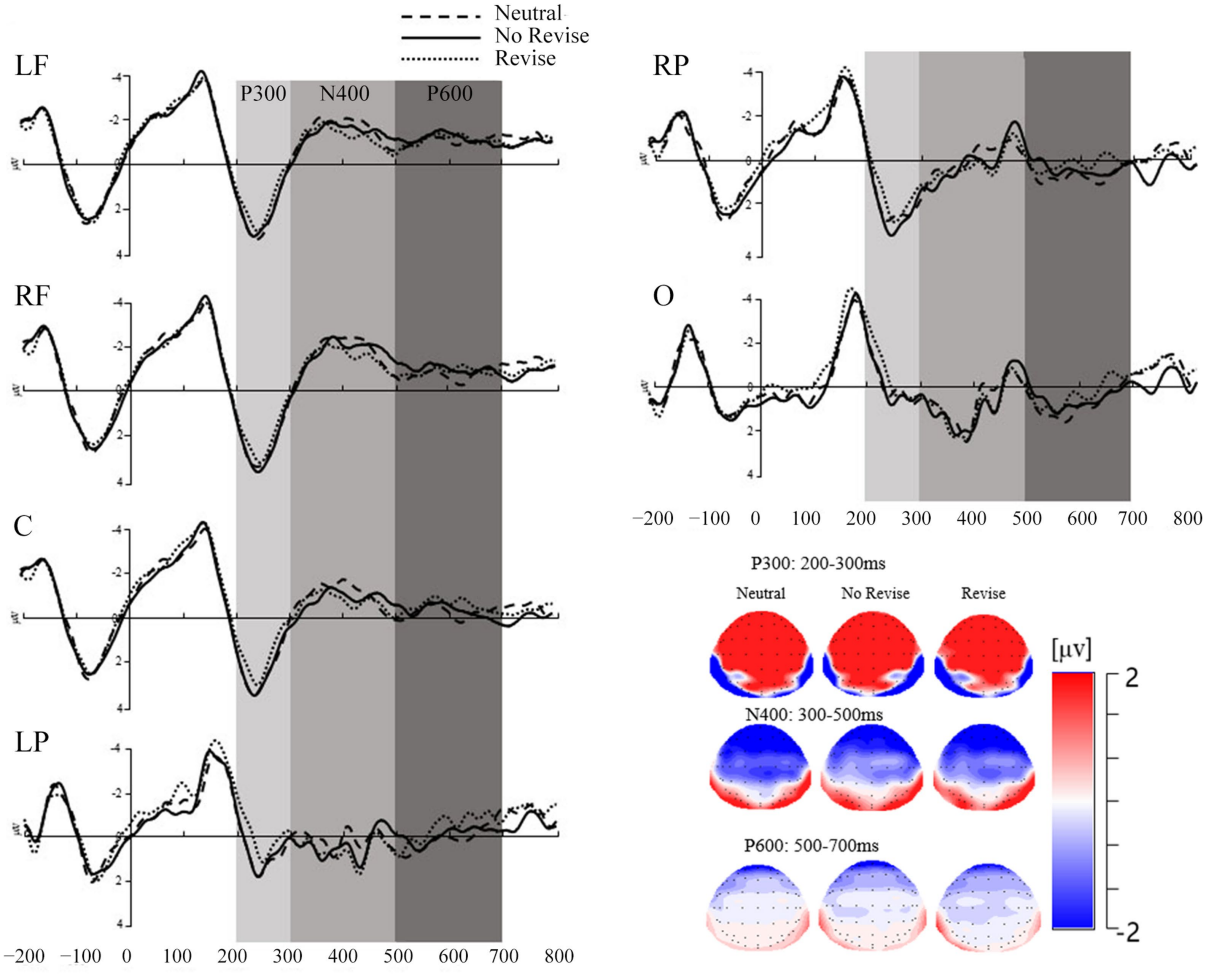
Graphical representation of the mean amplitude (in microvolts) for the P300, the N400, and the P600 components divided by contextual conditions and ROIs in Experiment 1.

The critical time windows were predefined by visual inspection of the grand averages and previous studies (e.g., Pérez et al., 2015, 2019). As a result, the mean amplitude was calculated in the time window of 200–300 ms for the ERP component of P3a and P3b and the time window of 300–500 ms for the ERP component of N400 after the disambiguating word onset (see Figure 2). Outlier amplitude data per condition, group and ROI were detected by the Box-Whisker plot and replaced by the mean for both the P300 (1.40%), the N400 (5.89%), and the P600 (2.43%).

### 2.2. Results

#### 2.2.1. Behavioral analysis

A one-way (contextual conditions: neutral, no revise, and revise) repeated measures ANOVA was conducted on the mean reading times for each condition. Table 2 presents the mean reading times for each condition both by subjects and by items. There was a main effect of contextual conditions, *F*_1_(2, 44) = 7.24, *p* = 0.002, *η*^2^_p_ = 0.25; *F*_2_(2, 178) = 3.98, *p* = 0.02, *η*^2^*_p_* = 0.04. Results of pairwise comparisons revealed that reading times in the revise condition (*M* = 1,461.96) significantly differed from either the neutral condition (*M* = 1,351.60), *p* = 0.04 and the no revise condition (*M* = 1,339.18), *p* = 0.005. There was no significant difference between the neutral condition and the no revise condition, *p* > 0.05.

**Table 2:** Descriptive statistics of reading time for the critical sentence in Experiment 1.

	By subjects		By items	
Condition	*M*	*SD*	*M*	*SD*
Neutral	1351.60	449.79	1344.44	357.49
No revise	1339.18	420.35	1331.86	343.08
Revise	1461.96	475.13	1462.50	370.90

Mean (*M*) and standard deviation (*SD*) values are shown.

#### 2.2.2. ERP analysis

The current study analyzed the amplitude of P3a and P3b in the time window of 200–300 ms through visual inspection. Since the P3a has been supposed to be more significant in the frontal areas while the P3b in the parietal areas. To test whether there were significant differences between the mean amplitudes of P3a and P3b in the data of the present study, a 3 (contextual conditions: neutral, no revise, and revise) × 6 (ROIs: central frontal: LF, RF, C, and posterior: LP, RP, and O) repeated measures ANOVA was conducted. Results showed a significant main effect of contextual conditions, *F*(2, 44) = 6.66, *p* = 0.003, *η*^2^_p_ = 0.23 with more positive amplitude in the neutral and no revise conditions. There was also a main effect of ROIs, *F*(5, 110) = 85.00, *p* < 0.001, *η*^2^_p_ = 0.79, with more positive amplitude in the central-frontal regions than the posterior regions. There was no significant interactive effect between contextual conditions and ROIs, *F*(10, 220) = 1.15, *p* = 0.33, *η*^2^_p_ = 0.05. Since there were significant differences between the mean amplitudes of P3a and P3b, the current study conducted separate analyses of the P3a in the central-frontal areas (LF, RF, and C) and the P3b in the posterior areas (LP, RP, and O).

#### 2.2.3. P3a analysis

It is assumed that readers could detect the mismatches in the critical sentence in the revise condition, update the current situation model with the alternative predictive inferences before considering the disambiguating word as having already been expressed implicitly in the critical sentence in the revise condition. In order to test this, a 3 (contextual conditions: neutral, no revise, and revise) × 3 (ROIs: LF, RF, and C) repeated measures ANOVA for P3a was conducted on the mean amplitude data for the disambiguating word in the time window of 200–300 ms. There was significant main effect of contextual conditions, *F*(2, 44) = 6.44, *p* = 0.004, *η*^2^_p_ = 0.23. The mean amplitude for P3a in the neutral condition (*M* = 3.30, *SD* = 1.72) and the no revise condition (*M* = 3.35, *SD* = 1.94) were significantly more positive than that in the revise condition (*M* = 2.66, *SD* = 1.92). There were no significant differences between the mean amplitude in the neutral and no revise conditions. The main effect of ROIs was also significant, *F*(2, 44) = 3.97, *p* = 0.03, *η*^2^_p_ = 0.15, with larger positivity in RF (*M* = 3.32, *SD* = 2.08) and in C (*M* = 3.08, *SD* = 1.93) than in LF (*M* = 2.89, *SD* = 1.55). There was no significant interaction between the contextual conditions and ROIs, *p* > 0.05.

#### 2.2.4. P3b analysis

A 3 (contextual conditions: neutral, no revise, and revise) × 3 (ROIs: LP, RP, and O) repeated measures ANOVA was conducted on P3b mean amplitude data in the 200–300 ms time window. There was a significant main effect of contextual conditions, *F*(2, 44) = 4.19, *p* = 0.02, *η*^2^_p_ = 0.16, with significantly more positivity in the neutral condition (*M* = 0.78, *SD* = 1.50) and the no revise condition (*M* = 0.74, *SD* = 1.44) than in the revise condition (*M* = 0.28, *SD* = 1.33). There were no significant differences between the mean amplitude in the neutral and no revise conditions. There was also a significant main effect of ROIs, *F*(2, 44) = 38.16, *p* < 0.001, *η*^2^_p_ = 0.63, with significantly more positivity in RP (*M* = 1.45, *SD* = 1.45) and LP (*M* = 0.41, *SD* = 1.24) than in O (*M* = −0.07, *SD* = 1.18). No significant interaction between the contextual conditions and ROIs was found.

#### 2.2.5. N400 analysis

A 3 (contextual conditions: neutral, no revise, and revise) × 6 (ROIs: central frontal: LF, RF, C, and posterior: LP, RP, and O) repeated measures ANOVA was conducted on the mean amplitude data of N400 in the time window of 300–500 ms. There was a significant main effect of contextual conditions, *F*(2, 44) = 5.91, *p* = 0.005, *η*^2^_p_ = 0.21, with significantly more negativity in the mean amplitude data in the neutral condition (*M* = −0.78, *SD* = 1.66) than in the revise condition (*M* = −0.45, *SD* = 1.56) than in the no revise condition (*M* = 0.01, *SD* = 1.47). There was also a significant main effect of ROIs, *F*(2, 44) = 30.11, *p* < 0.001, *η*^2^_p_ = 0.58, with the frontal-central areas, including LF (*M* = −1.11, *SD* = 1.71), RF (*M* = −1.37, *SD* = 2.02), and C (*M* = −0.69, *SD* = 1.45), being more negative than the parietal-occipital areas, namely LP (*M* = 0.24, *SD* = 0.99), RP (*M* = 0.01, *SD* = 1.07), and O (*M* = 0.48, *SD* = 1.11). A significant two-way interaction effect between contextual conditions and ROIs was also observed, *F*(2, 44) = 5.75, *p* < 0.001, *η*^2^_p_ = 0.21. Follow-up simple effects analysis showed a main effect of contextual constraints in the neutral condition, *F*(5, 110) = 27.65, *p* < 0.001 and the revise condition, *F*(5, 110) = 28.87, *p* < 0.001, showing more negativity in the amplitude data in the neutral and no revise condition than in the revise condition. In addition, the results also showed a main effect of ROIs in LF, *F*(2, 44) = 8.78, *p* = 0.001, RF, *F*(2, 44) = 7.51, *p* = 0.002, and C, *F*(2, 44) = 6.17, *p* = 0.004, indicating more negativity in these three ROIs than in the posterior and occipital regions.

#### 2.2.6. P600 analysis

A 3 (contextual conditions: neutral, no revise, and revise) × 6 (ROIs: central frontal: LF, RF, C, and posterior: LP, RP, and O) repeated measures ANOVA was conducted on the mean amplitude data of P600 in the time window of 500–700 ms. There was no significant main effect of contextual conditions. There was a significant main effect of ROIs, *F*(5, 110) = 17.49, *p* < 0.001, *η*^2^_p_ = 0.44, with the parietal-occipital areas, namely LP (*M* = −0.07, *SE* = 0.30), RP (*M* = 0.13, *SE* = 0.32), and O (*M* = 0.32, *SE* = 0.32) being more positive than the frontal-central areas including LF (*M* = −1.65, *SE* = 0.55), RF (*M* = −1.25, *SE* = 0.53), and C (*M* = −0.47, *SE* = 0.37). There was also a significant two-way interaction effect between contextual conditions and ROIs, *F*(10, 220) = 2.10, *p* = 0.03, *η*^2^_p_ = 0.09. Follow-up simple effects analysis showed a main effect of ROIs, indicating more negativity in frontal and central regions than in the posterior and occipital regions.

### 2.3. Discussion

#### 2.3.1. Mismatch evaluation

In Experiment 1, we exerted more efforts in ensuring higher contextual constraints for eliciting the primary predictive inferences. As a result, the primary predictive inferences have been supposed to be activated to greater levels, thus possibly more difficulties in disconfirming and revising them with inconsistent information. Results showed at least the following things. Firstly, behavioral results indicated that reading times for the critical sentence in the revise condition were significantly longer than that in the other two conditions. The finding corresponds to those studies that have found longer reading times when sentences contained contradictions with earlier statements (e.g., Myers et al., 1994; Albrecht and Myers, 1995). Secondly, ERP analysis showed that there was larger positivity in the mean amplitude data of P3a in the neutral and no revise conditions compared to the revise condition. The results perfectly replicate those of Pérez et al. (2015) and are consistent with the findings of successful mismatch detection (Potts et al., 1988; Nahatame, 2014).

The results concerning the P3a was convergent with the behavioral data to show at least two things. On the one hand, the significant difference between the revise condition and the neutral condition indicates that the preliminary predictive inferences have been activated and incorporated into participants’ situation model. On the other hand, this could also indicate that there is deactivation away from the now-inconsistent predictive inferences. And the alternative inferences have been activated to certain levels after reading the critical sentence in the revise condition.

Both the behavioral and electrophysiological results could be firstly explained by the Constructionist Theory (Graesser et al., 1994) that predictive inferences could be generated on-line if there is a high strength of activation from multiple information sources. This finding also corresponds to the Minimalist Hypothesis (McKoon and Ratcliff, 1992) in that predictive inferences could be automatically encoded if information is quickly and easily available from memory and when they are necessary to provide text coherence. Secondly, participants did detect the mismatches between the preliminary predictive inferences and the new incoming information. This shows that they have been engaged in an evaluation process. According to the Structure Building Framework (e.g., Gernsbacher, 1990), readers lay a foundation for their mental structures. The development of the foundation relies on a subsequent mapping on new information that is coherent with previous information. When incoming information is not coherent, a different process will appear and readers will shift to build new substructures. This process is resource-demanding.

#### 2.3.2. Disconfirmation of inconsistent predictive inferences

In Experiment 1, there was significantly larger positivity in the mean amplitude with P3b in the neutral and no revise conditions than in the revise condition. The results correspond partly to the results of Pérez et al. (2015) in which readers with high working memory exhibited less positivity in the revise condition. This result is also consistent with other previous studies showing that predictive inferences could be revised (e.g., Wright and Newhoff, 2002). The results suggest that participants could activate the alternative predictive inferences and disconfirm the primary predictive inferences when reading the critical sentence in the revise condition.

The assumption that primary predictive inference suppression and disconfirmation could happen might be supported by both the Structure Building Framework (Gernsbacher, 1990, 1997) and the KReC framework (Kendeou and O’Brien, 2014). According to the Structure Building Framework, once memory nodes have been activated, their activation levels are subject to two mechanisms, namely, suppression, and enhancement. Enhancement happens when the new incoming information has been considered necessary for building further structures. Suppression happens when the information has been considered inconsistent and irrelevant with the current memory representation (Gernsbacher, 1997). In Experiment 1, while participants were reading the critical sentence in the revise condition, they detected a mismatch between the contents in the current sentence and the previous information supplied in the introduction. Later, when reading the ERP sentence ended with a disambiguating word, they would consider the previously generated predictive inferences irrelevant and unnecessary for building a coherent situation model. Thus, the suppression and disconfirmation of the primary predictive inferences would happen in this situation to deactivate the previously generated but now outdated predictive inferences. While in the other two conditions, participants did not encounter any conflicts between predictive inferences generated from the previous introduction and the new information, and they could not disconfirm the inconsistent predictive inferences while reading the disambiguating word in the ERP sentence. The KReC framework suggests that when new information conflicts directly with information in the existing knowledge base, readers would update or revise the knowledge base to accommodate this newly encoded information. For the current study, the less positivity in the mean amplitude data of P3b in the revise condition shows that participants have disconfirmed the previously generated but now conflicting predictive inferences.

#### 2.3.3. Integration of alternative predictive inferences

Results concerning N400 in Experiment 1 could be elaborated from the following aspects. Firstly, significantly more negativity in the mean amplitude data in the neutral condition than in the revise condition and the no revise condition indicate that participants failed to integrate the alternative concepts in the revise condition. This is because the neutral condition is not related with either the initially generated predictive inference concepts or the alternatives and does not cause any semantic interferences. In this sense, there will be more cognitive resource consumption in the neutral condition compared with the revise condition, indicating that participants could integrate the alternative concepts to a certain extent. However, participants have difficulties integrating new information when it conflicts with the encoded information. Secondly, there were significant differences between the mean amplitude data in the revise condition and the no revise conditions, with the former being more negative. This shows a dominant position of the originally generated predictive inferences supported by strong contextual constraints in the introduction. The very strong initial contextual constraints provide a strong general context and could not be disconfirmed by insufficient local disconfirmation. More negativity observed in the neutral condition than in the no revise condition seems to reinforce the above-mentioned assumption that the very strong supporting contextual information for the initially made predictive inferences plays a predominant role in the reading comprehension.

In addition, according to the KReC framework (Kendeou and O’Brien, 2014), the revised information still exists in the long-term memory representation though it has lost activation and become less accessible on the comprehension of the subsequent text. However, they can still be reactivated and disrupt comprehension. This could explain why the integration of the revised predictive inference concepts is harder in the revise condition.

However, it is interesting to note that the integration of the revised predictive inference concepts is easier in the no revise condition of the critical sentences. This puzzling result might be explained by the fact that the current results put more emphasis on making sure of high contextual constraints of the preliminary predictive inference concepts, while neglecting the degree to which the contextual constraints support the alternative predictive inference concepts. The current experiment adopted more restrict methods to control contextual constraints of the introduction (Sentences 1–3) than in Pérez et al. (2015). In the latter, readers of high working memory capacity could revise the inconsistent predictive inferences and integrate the new information while those with low working memory capacities failed in both. This result could derive from the assumption that the already outdated predictive inferences has never been deleted from readers’ working memory and has the potential to be reactivated whenever necessary. Yet, these assumptions certainly await further investigation.

#### 2.3.4. Revision of inconsistent predictive inferences

Despite the absence of a significant main effect of contextual constraints, there was a significant main effect of ROIs and a significant interaction between contextual constraints and ROIs. The results indicate the influences of the ROIs on main effects of contextual constraints, which makes it hard for dissociating the effects of two factors on the revision process. This might indicate that participants in the no revise condition may still encounter difficulties in integrating the disambiguating word.

In sum, Experiment 1 managed to ensure considerably high contextual constraints for eliciting the primary predictive inferences. It aimed to explore whether participants could detect a mismatch between the primarily generated but now-inconsistent predictive inferences while reading the critical sentences in the revise condition. It also investigates whether readers could disconfirm and revise the inconsistent predictive inferences and integrate the alternative predictive inferences when reading the critical sentences in the revise condition. Both the behavioral results and mean amplitude data analysis of P3a suggests that participants could detect the mismatches through an evaluation process and disconfirm the incorrect predictive inferences. The above results perfectly replicate the results from other studies (e.g., Nahatame, 2014; Pérez et al., 2015) indicating that readers could detect the mismatches. However, the results of N400 and P600 mean amplitude data showed that readers had difficulties in either integrating the predictive inference alternatives or revising the primary predictive inferences. The results seem to support the assumption that the encoded predictive inferences still exist in the current working memory representation of readers, even though their activation levels have been reduced after reading the critical sentences in the revise condition. In addition, more restrict control of the contextual constraints in Experiment 1 than in other studies (e.g., Pérez et al., 2015, 2019) facilitate the activation of the primary predictive inferences, while reducing the activation levels of the alternatives. The discrepancies in findings of Experiment 1 and those of previous studies could be results of differences in the activation levels of alternatives. As a result, competitiveness of the primary predictive inferences and the alternatives should be the focus of further studies. Experiment 2 in the current study then tried to detect the effects of availability of weaker alternative predictive inferences on the predictive inference revision of the initially more strongly activated inferences.

One thing worth mentioning is that there are no significant differences between the neutral and no revise conditions in either the behavioral and ERP analyses. It is expected that there could be enhancement in the activation of the primary predictive inferences while reading the critical sentence in the no revise condition while no such enhancement should happen in the neutral condition. The activation degree for the primary predictive inferences should be quite different in these two conditions. Accordingly, the mismatches between the disambiguating words in the ERP sentences and the critical sentences in these two conditions should be different. The mean amplitude data of P3a should be larger in the no revise condition than in the neutral condition. However, no such significant differences were observed in Experiment 1. It is assumed, therefore, the activation levels of the primary predictive inferences should be quite similar in these two conditions. And the differences in these two conditions and the revise condition, being significant, are more worthy of further investigation. Experiment 2 then focuses on the study of predictive inference revision process with the availability of weakly activated alternatives in the two conditions, namely, the revise and no revise conditions.

## 3. Experiment 2

### 3.1. Method

#### 3.1.1. Participants

Thirty-two Chinese native speakers from a university were paid for participating in the experiment. All had normal or corrected-to-normal vision, and none had any language disorder, neurological disorder, or major head injury diagnosed to have long-term side effects. All gave informed consent before participation. Data from one participant were discarded due to technical failures and one due to blinking artifacts. The remaining 30 participants (10 males and 20 females, *M*_age_ = 21.27, range 17–26, *SD* = 2.88) were included for analysis. All participants were right-handed as assessed by the Edinburgh Handedness Inventory (Oldfield, 1971), with a mean laterality of 0.88 (range 0.65–1, *SD* = 11.55) indicating right-handedness. Participants were also engaged in a conventional Digit Span Forward (*M* = 9.73, range 8–12, *SD* = 0.98) with a total score of 12, and Backward (*M* = 7.10, range 4–10, *SD* = 1.71) with a total score of 10 for assessing working memory (Gong, 1983), indicating high levels of working memory for participants.

#### 3.1.2. Materials

Experiment 2 conducted a norming study including both a story continuation procedure (see Estevez and Calvo, 2000; Cook et al., 2001) and a rating task (see Cook et al., 2001; Cook and O’Brien, 2014; Cranford and Moss, 2019) to choose the 3-sentence passages whose contextual constraints were strong enough to induce two plausible predictive inferences, with one having greater activation levels and the other one containing weaker activation levels. A total of 192 short 3-sentence Chinese narratives were created with strong contextual constraints. These short narratives were based on typical, everyday events. From these 192 short passages, 63 (three practice and 60 experimental) were rated as having strong contextual constraints which induced a main predictive inference and a weaker alternative. There were two parts in the norming study of Experiment 2. The first part consisted of a cloze procedure that is the same with that in Experiment 1. For the second part of the norming study, participants were requested to rate the probability of the occurrence on a 7-point Likert-type Scale ranging from 1 (extremely unlikely) to 7 (extremely likely) with 4 being neutral, immediately after the two verbs had been provided. The first most frequently expected words derived from the introduction and the most highly rated ones were selected as the primary target words, representing the more strongly activated primary predictive inferences. In addition, the second most frequently expected words derived from the introduction and the secondary highly rated words were decided as the alternative target words representing the low competitive alternative predictive inferences. Sixty passages were chosen out of the 192 based on the following two criteria: more than 80% of the participants should supply the same first target words and lower than 30% with the second target words or their closest synonyms according to The Modern Chinese Standardized Dictionary (Li, 2014). After the 60 passages had been chosen, the rating results of the two targets in them were then subject to further analyses. Results of the rating task for the two target words showed that there was significant difference between the rating of the first (*M* = 5.81, *SD* = 0.30) and the second target words (*M* = 5.01, *SD* = 0.35), *t*(59) = 13.36, *SD* = 0.47, *p* < 0.001. The rating result indicated that the first target word was more possible to happen next. It followed naturally from the norming study results that the introduction (Sentences 1–3) strongly constrained for the first target word while being weakly constrained for the second target word. A sample material is shown in Table 3.

**Table 3:** Sample material used in Experiment 2.

Introduction	bias 抄袭(*Chāo Xí*)	小明通过这次考试才能毕业。 他看到最后一个题目就蒙了。 他偷偷地瞥了监考老师一眼。
	*English translation* bias *plagiarizing*	*English translation*
		Xiaoming could graduate only if he passed this examination.
		He was puzzled at the sight of the last question.
		He peeped at the supervisors of the examination.
No revise	他能看清楚旁边考生的答案。	
	*English translation*	
	He could see his neighbor’s answers clearly.	
Revise	他收拾好试卷走向监考老师。	
	*English translation*	
	He took up his answer sheet and walked to the supervisors.	
ERP sentence	他实在是不会做就打算交卷(*Jiāo Juàn*)。	
	*English translation*	
	Unable to answer any more questions, he decided to *submit* the answer sheet.	
Comprehension question	他看到最后一个题目蒙了吗?	
	*English translation*	
	Was he puzzled at the sight of the last question?	

As stated above, there were no significant differences in either the behavioral and ERP analyses for the neutral and no revise conditions and we are more concerned with the differences between the revise and no revise conditions. Therefore, there were only two versions of the critical sentence, i.e., the revise and no revise conditions in Experiment 2.

The no revise version was consistent with the inferences primed by the introduction. The revise version contained information that was inconsistent with the initial predictive inferences and prompted participants to revise the inconsistent predictive inferences. Consistent with the measures of Experiment 1, reading times for the critical sentence was also recorded. ERPs were recorded at the disambiguating word at the very end. Following each narrative text, a comprehension question with the same design and purposes with that in Experiment 1 was also presented. Each of the five sentences in every version also contained 12 Chinese characters to avoid extra horizontal electrooculogram activities and artifacts.

#### 3.1.3. Procedure

Experiment 2 followed a similar procedure with that of Experiment 1, with the exception that the ERP sentence was presented word by word with a fixed SOA of 300 ms per word, with an interval of 500 ms between two words – a longer time duration than that in Experiment 1 to maintain a more stable baseline. There was still a delay of 700 ms after the ERP word to ensure the recording of electrophysiological activities during a sufficiently long time-window (SOA = 1,000 ms).

The 60 sets of experimental texts (in two conditions) were assigned into two versions. Each of the 60 experimental texts of a version was presented to each participant only once in one of the two conditions which had been counterbalanced across participants. The task was administered in three blocks, keeping 10 texts in each condition per block (altogether three blocks). The whole procedure lasted for about 45 min. Experiment 2 followed the same apparatus settings with Experiment 1 except for the fact that trials with artifacts were rejected (1.67%) with potentials exceeding ±100 μv.

#### 3.1.4. Statistical analysis

We report statistical analyses of 30 participants for all trials. The results of T-test comparison on reading times of the critical sentences showed that there were no differences between the sample with correct responses only and the whole sample, *t*(29) = −0.27, *p* = 0.79. As a result, both correct and incorrect responses were also included for analysis for both behavioral and electrophysiological analyses.

The behavioral analysis of the predictive inference revision task followed the same procedure in Experiment 1. Accuracy was also quite high in both the no revise condition (*M* = 0.95, *SD* = 0.04) and the revise condition (*M* = 0.95, *SD* = 0.05; averaging at 95.05%, range 83–100%), close to ceiling and not significantly differing across conditions or participants [*t*(29) = 0.77, *p* = 0.45].

**Figure 3:**
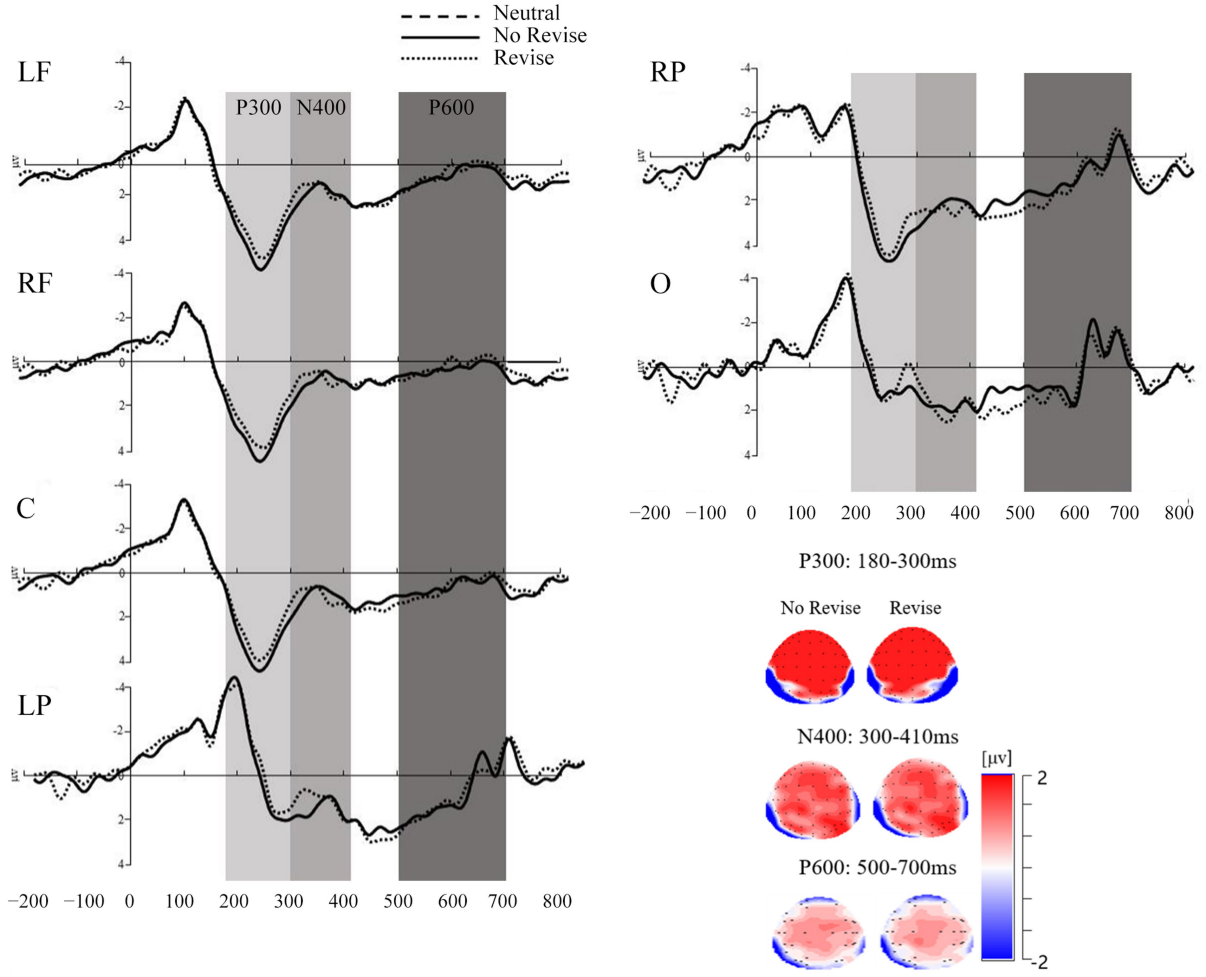
Graphical representation of the mean amplitude (in microvolts) for the P300, the N400, and the P600 components divided by contextual conditions and ROIs in Experiment 2.

For the ERP analyses, the critical time windows were predefined by visual inspection and previous studies (e.g., Pérez et al., 2015, 2019). As a result, the mean amplitude was calculated in the time window of 180–300 ms for the ERP components of P3a and P3b, the time window of 300–410 ms for the ERP component of N400, and the time window of 500–700 ms for P600 after the disambiguating word onset (see Figure 3). Outlier amplitude data per condition, group and ROI were detected by the Box-Whisker plot and replaced by the mean for both the P300 (3.33%), the N400 (2.80%), and the P600 (1.31%).

### 3.2. Results

#### 3.2.1. Behavioral analysis

T-tests showed significant differences between the no revise (*M* = 1713.47, *SD* = 666.15) and revise conditions (*M* = 1917.90, *SD* = 663.07), *t*(29) = −5.77, *p* < 0.001.

#### 3.2.2. ERP analysis

A 2 (contextual conditions: no revise and revise) × 6 (ROIs: central frontal: LF, RF, C, and posterior: LP, RP, and O) repeated measures ANOVA was carried out to see whether there were significant differences between the mean amplitudes of P3a and P3b in the data of Experiment 2. Results showed a significant main effect of contextual conditions, *F*(1, 29) = 6.13, *p* = 0.02, *η*^2^_p_ = 0.18, with more positive amplitude in the no revise condition (*M* = 2.45, *SD* = 3.04) than the revise condition (*M* = 1.97, *SD* = 2.88). There was also a main effect of ROIs, *F*(5, 145) = 44.11, *p* < 0.001, *η*^2^_p_ = 0.60, with more positive amplitude in the central-frontal regions than the posterior regions. A follow-up repeated measures ANOVA was conducted on mean amplitudes in different ROIs (central frontal: LF, RF, C, and posterior: LP, RP, and O), *F*(5, 295) = 47.29, *p* < 0.001, *η*^2^_p_ = 0.45, with the mean amplitude more positive in central frontal areas, specifically, LF (*M* = 3.20, *SD* = 2.08), RF (*M* = 4.00, *SD* = 2.54), and C (*M* = 3.63, *SD* = 2.75) than in the posterior areas, specifically LP (*M* = 0.42, *SD* = 2.61), RP (*M* = 2.02, *SD* = 2.80), and O (*M* = −0.01, *SD* = 2.42). There was no significant interactive effect between contextual conditions and ROIs, *F*(5, 145) = 2.11, *p* = 0.07, *η*^2^_p_ = 0.07. Since there were significant differences between the mean amplitudes of P3a and P3b, Experiment 2 also carried out separate analyses of the P3a in the central-frontal areas (LF, RF, and C) and the P3b in the posterior areas (LP, RP, and O).

#### 3.2.3. P3a analysis

A 2 (contextual conditions: no revise and revise) × 3 (ROIs: LF, RF, and C) repeated measures ANOVA was conducted on the mean amplitude data with P3a for the disambiguating word in the time window of 180–300 ms. There was significant main effect of contextual conditions, *F*(1, 29) = 6.73, *p* = 0.02, *η*^2^_p_ = 0.19. The mean amplitude data with P3a in the no revise condition (*M* = 3.91, *SD* = 2.64) were significantly more positive than those in the revise condition (*M* = 3.30, *SD* = 2.28). The main effect of ROI was also significant, *F*(2, 118) = 10.49, *p* < 0.001, *η*^2^_p_ = 0.15, with larger positivity in RF (*M* = 4.00, *SD* = 2.54) and in C (*M* = 3.63, *SD* = 2.75) than in LF (*M* = 3.20, *SD* = 2.08). There was no significant interaction between the contextual conditions and ROIs, *F*(2, 58) = 0.50, *p* = 0.61, *η*^2^_p_ = 0.02.

#### 3.2.4. P3b analysis

A 2 (contextual conditions: no revise and revise) × 3 (ROIs: LP, RP, and O) repeated measures ANOVA was conducted on the mean amplitude data with P3b in the 180–300 ms time window. There was a significant main effect of contextual conditions, *F*(1, 29) = 4.32, *p* = 0.05, *η*^2^_p_ = 0.13, with significantly more positivity in the no revise condition (*M* = 1.00, *SD* = 2.69) than in the revise condition (*M* = 0.63, *SD* = 2.79). There was also a significant main effect of ROIs, *F*(2, 118) = 64.12, *p* < 0.001, *η*^2^_p_ = 0.52, with significantly more positivity in RP (*M* = 2.02, *SD* = 2.80) and the LP (*M* = 0.42, *SD* = 2.61) than in O (*M* = −0.10, *SD* = 2.42). No significant interaction between the contextual conditions and ROIs was found, *F*(2, 58) = 2.18, *p* = 0.12, *η*^2^_p_ = 0.07.

#### 3.2.5. N400 analysis

A 2 (contextual conditions: no revise and revise) × 6 (ROIs: LF, RF, C, LP, RP, and O) repeated measures ANOVA was conducted on mean amplitude data with N400 in the time window of 300–410 ms. There was no significant main effect of contextual conditions, *F*(1, 29) = 1.22, *p* = 0.28, *η*^2^_p_ = 0.04. There was a significant main effect of ROIs, *F*(5, 145) = 2.72, *p* = 0.02, *η*^2^_p_ = 0.09, with the frontal areas including LF (*M* = 1.00, *SD* = 1.84), RF (*M* = 0.86, *SD* = 1.79), and O (*M* = 0.75, *SD* = 1.25) having smaller amplitude than C (*M* = 1.25, *SD* = 1.98), and the parietal areas, namely, LP (*M* = 1.22, *SD* = 1.72), and RP (*M* = 1.58, *SD* = 1.74). There was no significant two-way interaction effect between contextual conditions and ROIs, *F*(5, 145) = 1.80, *p* = 0.12, *η*^2^_p_ = 0.06.

#### 3.2.6. P600 analysis

A 3 (contextual conditions: neutral, no revise, and revise) × 6 (ROIs: central frontal: LF, RF, C, and posterior: LP, RP, and O) repeated measures ANOVA was conducted on the mean amplitude data of P600 in the time window of 500–700 ms. There was no significant main effect of contextual conditions. There was a significant main effect of ROIs, *F*(5, 145) = 4.14, *p* = 0.002, *η*^2^_p_ = 0.13, with the central-parietal areas, namely C (*M* = −0.71, *SE* = 0.23), LP (*M* = 0.22, *SE* = 0.20), and RP (*M* = 0.36, *SE* = 0.18) being more positive than the frontal-occipital areas including LF (*M* = 0.10, *SE* = 0.23), RF (*M* = 0.22, *SE* = 0.25), and O (*M* = −0.06, *SE* = 0.16). There was no significant two-way interaction effect between contextual conditions and ROIs.

### 3.3. Discussion

Both the study from Pérez et al. (2015, 2019) and Experiment 1 of the current study failed to be concerned about the influences of a weakly activated alternative predictive inference that could be generated together with the preliminary predictive inferences. Like the dominant predictive inferences, the alternative predictive inferences could also be generated and encoded into readers’ working memory. Once the weaker alternatives remained in readers’ memory representation, they brought influences to the dominant one. Despite its weaker activation, they could still draw activation away from the once strongly activated predictive inferences to themselves. Experiment 2 attempted to discover the potential effects of weaker alternative predictive inferences on the revision process while maintaining a high contextual constraint for the primary ones.

#### 3.3.1. Mismatch evaluation

Consistent with results of previous studies and those of Experiment 1, the longer reading time and reduction in mean amplitude data of the P3a in the revision condition of Experiment 2 would indicate the cognitive resource consumption in detecting such a mismatch.

#### 3.3.2. Disconfirmation of inconsistent predictive inferences

In Experiment 2, there was significantly larger positivity of mean amplitude of P3b in the no revise condition than the revise condition, which suggested that readers could disconfirm the initially strong predictive inferences with the weaker ones while reading the critical sentence in the revise condition. The results aligned with the findings from Experiment 1 of our current study and previous studies in that predictive inferences could be suppressed (e.g., Fincher-Kiefer, 1995; Klin et al., 1999b; Iseki, 2006; Pérez et al., 2015) and disconfirmed when inconsistent information followed (e.g., Wright and Newhoff, 2002; Nahatame, 2014; Pérez et al., 2015, 2019).

#### 3.3.3. Integration of alternative predictive inferences

For the mean amplitude data of N400, there was no significant difference in the revise condition and the no revise condition. This result differs from the findings of Pérez et al. (2015, 2019) and the findings in our Experiment 1. The lack of significant differences could be explained by relevant proposals in the Resonance Model (Myers et al., 1994; Myers and O’Brien, 1998). Results of Experiment 2 reveals that readers could not successfully integrate an initially weakly activated but later-to-be-revised predictive inferences immediately following the information supporting it. This might indicate that the very strong initial contextual constraint provides a strong general context and could not be disconfirmed by insufficient local disconfirmation.

#### 3.3.4. Revision of inconsistent predictive inferences

There was no significant difference for P600 data in the two conditions, either. The lack of significant differences in the mean amplitude data of P600 in the two conditions showed that the originally dominant predictive inferences, though disconfirmed, remained to be active in readers’ memory representation. The results seem also to support the assumption that the already-encoded predictive inferences still exist in the current working memory representation of readers’, even though their activation levels have been reduced after reading the critical sentence in the revise condition.

## 4. General discussion

For materials used in both Experiment 1 and Experiment 2, there is a strong contextual constraint for eliciting the primary predictive inferences. As a result, it is contextually insufficient for the activation and encoding of the alternative predictive inferences. However, Experiment 1 and prior research into predictive inference revision paid little attention to the influences of the activation levels of alternative predictive inferences, even though there was stricter control of contextual constraints for eliciting the primary predictive inferences. Experiment 1 failed to replicate the findings of previous studies (e.g., Pérez et al., 2015), which had discovered successful revision of the incorrect predictive inferences and integrating the alternative ones for readers of high working memory capacity. However, as previously stated, we hold doubts about the reliability of rendering the ERP component of P3b as an index of predictive inference revision. P3b tends to be rendered as an index reflexive of the disconfirmation of an expectation. As a result, despite the same findings in Experiment 1 with those of the previous studies, we are doubtful about readers’ successful revision. In addition, the lack of significant differences in the N400 shows readers’ failure hints the lingering interruption of the primary predictive inferences. Experiment 2 in the current study then adopted restricted methods to control contextual constraints of the introduction as prompting more dominant primary predictive inferences and weaker alternatives. Results showed that participants could carry out mismatch detection and disconfirm the primary predictive inferences that were contradicted by new incoming information. However, they also had difficulties in either integrating the alternative inferences or revising the primary predictive inferences.

The results could be explained in the following ways. Firstly, a weakly constrained predictive inference alternative can hardly compete with a strongly activated predictive inference in the first place. The weak competitiveness lingers on after the primarily strong predictive inferences have been disconfirmed. Secondly, it is possible that activation of a specific predictive inference decays to be a more general one with delay, and readers are left with the instantiation of a more general prediction for both alternative predictive inferences. Failure of integrating the alternative predictive inferences indicates that it is still considered not closely related to the current situation model. And revision of incorrect predictive inferences would happen when sufficient amount of supportive information comes in to back up the alternative ones. This is in accordance with the proposal that knowledge revision happens only after the amount and quality of information integrated into the knowledge base has crossed certain threshold could knowledge revision become evident (Kendeou and O’Brien, 2014, 353). As it is held by the KReC framework, integrating new information during reading results in the updating or revision of the emerging discourse representation. If new information is not integrated with the already-acquired information, revision has not occurred (Kendeou and O’Brien, 2014).

Prior studies have found that readers could hardly remove the incorrect information form their working memory (e.g., Wilkes and Leatherbarrow, 1988; Johnson and Seifert, 1994; Nahatame, 2014). The outdated information might be reactivated because the disconfirmed information remains in the working memory and would return to the memory representation whenever supported by the incoming information (e.g., Campion, 2004; Guéraud et al., 2005). In Experiment 2, there was little competition between the alternative predictive inferences elicited from the introduction. The initially generated predictive inferences held a strong activation level by a strong contextual constraint. As a result, it would be less possible to eliminate the originally dominant predictive inferences from the working memory representation than in Experiment 1 and previous studies (e.g., Pérez et al., 2015, 2019). This could be supported by the findings from Weingartner et al. (2003) which found that the targeted inference was not deleted in the presence of alternative consequences.

To conclude, the present study assumed the role of the first attempt to detect the effects of predictive inference alternatives of weaker activation levels as compared to the primary inferences under high contextual constraints. The findings from our experiments suggest that readers could almost automatically detect mismatch information against the primary predictive inferences through an evaluation process. They could also suppress and disconfirm the inconsistent primary predictive inferences. However, they have difficulties in either integrating the alternative predictive inferences of weaker activation levels or revising the consistent primary predictive inferences. The findings suggest that the primary predictive inferences, though having been disconfirmed, still maintain their activation levels and could be reactivated any time when new information comes in to provide further support and possibly exert influences on both the integration of the alternative predictive inferences and revision of the inconsistent ones. Our findings are supportive of the KReC framework in that once the information has been encoded into readers’ working memory, they could hardly be deleted and could be reactivated whenever they are supported by sufficient amount of new information. Our findings also demonstrate that the revision process is a slow, incremental and conservative one which involves the co-activation and competing activation between the outdated predictive inferences and the alternative ones. Future studies could further study whether the inconsistent, but primarily more strongly generated predictive inferences would be deleted and totally replaced by the alterative predictive inferences. Moreover, whether predictive inference revision happens when the alternative predictive inferences are of almost equal activation levels to the primary predictive inferences is also worthy of further investigation.

## Data availability statement

The raw data supporting the conclusions of this article will be made available by the authors on request, without undue reservation.

## Ethics statement

The studies involving human participants were reviewed and approved by Beijing Foreign Studies University. The patients/participants provided their written informed consent to participate in this study.

## Author contributions

FX, LF, and LT designed the two experiments. LF provided laboratory support. FX and LT carried out the experiments. FX and LT analyzed the data and wrote the draft manuscript. LF and LC provided data analysis methods and edited the manuscript. All authors contributed to the article and approved the submitted version.

## Funding

This work was supported by research grants awarded by National Planning Office of Philosophy and Social Science (18BYY088), Humanities and Social Sciences Research Funds Project of Qingdao University of Technology (Year 2022) (Crw2022-019), Undergraduate Teaching Reform and Research Project of Qingdao University of Technology (Year 2022) (W2022-056), Ministry of Education Humanities and Social Sciences Research Youth Fund Project of the People’s Republic of China (20YJC740008), the Fundamental Research Funds for the Central Universities (22CX04014B), and the 71st Batch of China Postdoctoral Science Foundation (2022M712151).

## Conflict of interest

The authors declare that the research was conducted in the absence of any commercial or financial relationships that could be construed as a potential conflict of interest.

## Publisher’s note

All claims expressed in this article are solely those of the authors and do not necessarily represent those of their affiliated organizations, or those of the publisher, the editors and the reviewers. Any product that may be evaluated in this article, or claim that may be made by its manufacturer, is not guaranteed or endorsed by the publisher.
